# Integrated hierarchical surface restructuring of assembled electrode arrays for next-generation neural interfaces

**DOI:** 10.1371/journal.pone.0348879

**Published:** 2026-06-02

**Authors:** Alexander Blagojevic, Wesley Roser, Wesley Seche, Pouya Tavousi, Sina Shahbazmohamadi, Shahram Amini

**Affiliations:** 1 Department of Biomedical Engineering, University of Connecticut, Storrs, Connecticut, United States of America; 2 Pulse Technologies Inc. (An Integer Holdings Company), Research and Development, Quakertown, Pennsylvania, United States of America; 3 Tescan Orsay Holding, Brno-Kohoutovice, Czechia; Mustansiriyah University, IRAQ

## Abstract

Neurostimulation devices rely on electrode arrays to deliver targeted electrical stimulation for modulating nerve activity. Enhancing stimulation specificity, device battery and energy efficiency, and device miniaturization requires low-profile electrodes with exceptional electrochemical performance. Hierarchical Surface Restructuring (HSR™), a femtosecond laser-based electrode surface treatment technology, enables these improvements by significantly increasing the electrochemically active surface area of the electrode contacts through the formation of highly textured, multi-scale architectures. Although HSR™ offers substantial potential to enable both high-performance electrodes and further miniaturization of electrode arrays, its broader adoption in medical device manufacturing has been limited by cost considerations and the inherent complexities of integrating new surface modification steps into established production workflows. This study investigates the feasibility of applying HSR™ technology to commercially available Pt-10Ir paddle-type electrode arrays and, for the first time, demonstrates that HSR™ can be implemented as a stand-alone, post-fabrication surface modification process that is compatible with existing device geometries and material constraints. This advancement represents a significant step toward broader adoption of HSR™ by medical device manufacturers and demonstrates its overall manufacturing viability. The process developed in this study circumvents key barriers to industrial implementation by enabling HSR™ to be seamlessly integrated into existing production lines as a post-fabrication surface modification step, thereby eliminating the need for major or costly process changes. The morphology, electrochemical performance, and processing efficiency of the restructured electrodes were systematically characterized. HSR™ enhanced key electrochemical metrics—including charge storage capacity, specific capacitance, and impedance—by up to two orders of magnitude, while maintaining short processing times and full compatibility with the device’s geometry and constituent materials. These findings demonstrate the potential for HSR™ to be seamlessly integrated into existing manufacturing workflows as a post-fabrication step, providing a scalable and cost-effective approach for enhancing the electrochemical performance of neurostimulation electrode arrays. Furthermore, in-operando CO_2_-snow-assisted processing was shown to be equally compatible with established production lines, improving electrode stability and surface cleanliness without necessitating any upstream process modifications.

## Introduction

Neurostimulation technologies have emerged as powerful therapeutic modalities for treating a wide range of neurological and neuromuscular disorders, including chronic pain, motor dysfunction, and peripheral neuropathies [1–3]. These therapies rely on implantable electrode arrays that deliver targeted electrical stimulation to modulate neural activity within specific regions of the nervous system. Among the many applications of neurostimulation, spinal cord stimulation (SCS) is one of the most clinically established. In SCS, electrode arrays are typically implanted in the epidural space of the spine, where they interface with spinal neurons to activate or suppress neural pathways involved in motor function and pain processing [3–6].

**Fig 1:**
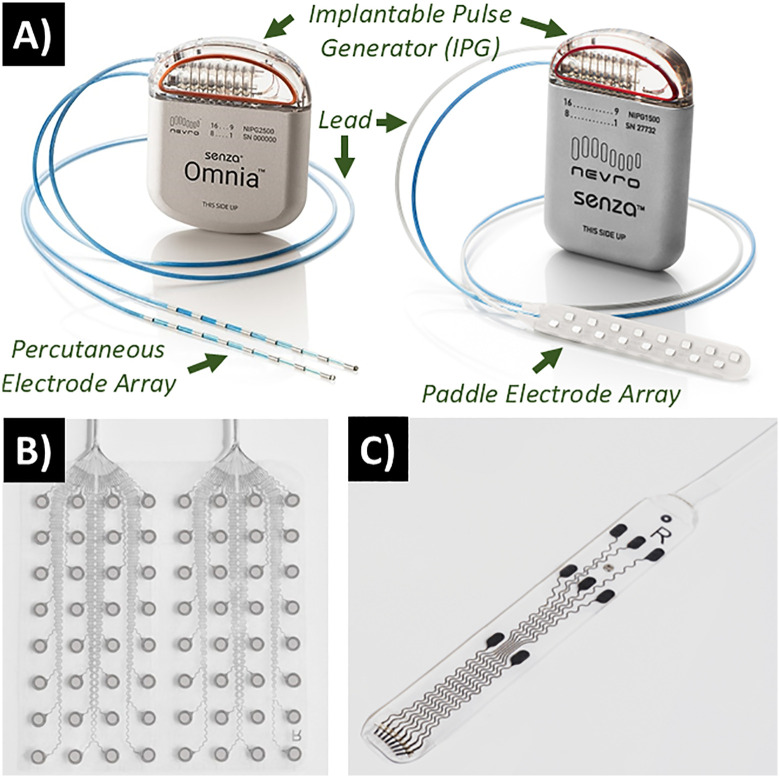
Examples of various geometries and arrangements of commercially available (micro)electrode arrays. **A)** Nevro® Senza® spinal cord stimulators with percutaneous lead-type (left) and paddle-type (right) arrays. **B)** CorTec® °AirRay® cortical grid microelectrode array used for ECoG applications; **C)** CorTec® °AirRay® paddle microelectrode array used for spinal cord stimulation. All images used with permission and courtesy of Nevro Corp and CorTec®.

Spinal cord electrode arrays are typically categorized into two types: cylindrical (percutaneous), or flat (paddle), as shown in Fig 1a. Percutaneous arrays offer a minimally invasive and low-risk device profile with easy adjustability, but their use is limited by reduced spatial coverage, reduced energy efficiency, and a higher risk of electrode migration over time [7,8]. Paddle-type electrode arrays offer greater energy efficiency and spatial selectivity, but their main drawback is a more invasive device profile [8,9]. Both types of spinal cord electrode arrays are effective in providing substantial pain relief, but paddle-type arrays are more commonly employed in advanced therapies and research applications beyond basic pain management [8,10,11]. Therefore, there is a growing need for advanced electrode manufacturing techniques that enable further miniaturization of paddle-type electrode arrays to reduce their invasiveness—an aspect that not only increases surgical complexity and patient risk, but can also compromise long-term device performance and stability [12].

As with most neural interfacing applications, ideal electrode arrays for SCS are those that exhibit a high electrochemically active surface area (ESA) relative to their geometric surface area (GSA). Such high-performance electrodes are characterized by low impedance, high charge storage and injection capacities (CSC and CIC), high specific capacitance, and improved energy efficiency [13–15]. These properties enable the fabrication of high-density electrode arrays which allow more electrodes to interface with the target tissue, improving spatial resolution and selective targeting, allowing for more precise modulation or monitoring of biological functions [12–16].

While these outcomes are desirable for many neurostimulation applications, the reduction in GSA required to achieve miniaturized and high-density electrode array configurations can also limit the total charge that each electrode contact can deliver, therefore compromising energy efficiency, signal-to-noise ratio, and overall device functionality. The inherent constraints of conventional manufacturing techniques limit the extent to which electrode arrays can be further miniaturized, without compromising functional electrochemical performance [17]. Therefore, the next-generation of neurostimulation devices will rely on materials with a high ESA to GSA ratio, facilitating the fabrication of miniaturized, high-performance electrode contacts and densely packed electrode arrays [13,14,18].

To overcome the tradeoff between geometric surface area (GSA) and electrochemical performance, various surface modification techniques have been developed to increase the electrodes’ ESA, independently of GSA. Amongst the most widely used are thin films and coatings of highly conductive materials such as iridium oxide (IrO_2_) [19–22], titanium nitride (TiN) [19,23–25], two-dimensional materials [26,27], and other electrochemically active thin films [28]. Recently, femtosecond laser-based Hierarchical Surface Restructuring (HSR™) has emerged as a novel surface engineering approach capable of dramatically enhancing the electrochemical performance of platinum-iridium (Pt-10Ir) electrodes [29–32]. This technique utilizes a femtosecond laser beam to create periodic arrays of mound-shaped hierarchical structures on an electrode surface, characterized by levels of topographies that span various length scales.

These hierarchical structures substantially increase the ESA independently of GSA, which is an important characteristic for electrode miniaturization while maintaining high electrochemical performance. Prior studies have demonstrated that HSR™ can increase the charge storage capacity of Pt-10Ir electrodes by up to two orders of magnitude and the specific capacitance by more than 700-fold [29,30]. The morphology and electrochemical performance of HSR™ treated electrodes are highly tunable via laser parameters. The technique can be applied on electrodes as thin as 20 µm [29,30]. Because HSR™ is a precisely targeted ablative process, it does not require additional materials in the form of coatings or masks, and circumvents issues related to coating overspray and delamination [18]. This substantially reduces fabrication time and cost, making the technique well-suited for scalable, serial production.

### Objectives

Although HSR™ has demonstrated strong potential to simultaneously enhance the performance and enable the miniaturization of neurostimulation electrodes, its adoption by medical device manufacturers has remained limited. As with many emerging manufacturing technologies, implementation is often constrained by the cost and complexity associated with modifying or replacing established production processes. In a brief case study reported in a previous publication [30], it was demonstrated that HSR™ technology can be adapted to the unique material and geometric constraints of implantable neurostimulation electrodes, such as the cortical grid arrays shown in Fig 1b and 1c. Notably, the process was successfully applied to fully assembled electrode arrays, preserving the integrity of the existing manufacturing process without requiring disassembly or redesign. However, this proof-of-concept study utilized only a single set of laser parameters, leaving a vast design space unexplored, with respect to performance optimization and more complex device geometries.

In this study, we aim to further demonstrate the feasibility and tunability of femtosecond laser HSR™ on prefabricated, fully assembled commercially available electrode arrays by applying the process to a Pt-10Ir paddle-type electrode array without requiring any modifications to the existing manufacturing process. Specifically, we seek to evaluate the practical implementation of HSR™ in terms of processing speed, material compatibility, and geometric conformity with the array’s existing design. Additionally, the study will assess the electrochemical performance enhancements achievable through this approach, focusing on relevant electrochemical performance metrics critical to neural interfacing applications.

Unlike the previous case study, which focused on a relatively flat cortical grid electrode array, the present arrays feature a more complex geometry which introduces new processing challenges. The arrays have a semicircular cross-section, making it difficult to mount and level for accurate and consistent targeting under the laser system. Moreover, the electrode contacts themselves are not uniformly level, presenting variations in height that can affect laser focal precision. These geometric discrepancies must be carefully accounted for to maintain proper laser focus across the entire surface of the electrode contacts, ensuring uniform restructuring and consistent electrochemical performance.

As reported previously [29,30], the performance of HSR™-treated electrodes can be systematically tuned by modifying key laser processing parameters, enabling application-specific optimization. For instance, increasing average power—and thus laser fluence—has been shown to enhance electrochemical performance metrics. However, this also leads to a larger effective spot size and greater thermal energy deposition on the surface, elevating the risk of collateral damage to adjacent regions, particularly the polymer insulation surrounding the electrode contacts [29]. Therefore, a key objective of this study is to investigate a range of laser fluence levels to determine the safe operational window for restructuring paddle-type arrays. This includes identifying fluence thresholds that yield significant performance improvements, while preserving the structural and functional integrity of the array’s surrounding components.

Furthermore, this study aims to evaluate the feasibility of integrating CO_2_ snow processing with femtosecond laser HSR™ treatment on fully assembled electrode arrays. CO_2_ snow processing is a non-contact, in-operando surface preparation technique wherein gaseous carbon dioxide (CO_2_) is compressed to its triple point and expelled through a nozzle, which is directed precisely at the laser’s focal point. This method effectively dislodges particulates, surface debris, and contaminants during the laser process, without leaving behind residues or causing significant mechanical abrasion. Importantly, it preserves the underlying electrode morphology and ensures a cleaner, more stable surface [33,34]. This objective is critical for validating a scalable, inline-compatible post-processing solution that maintains the performance and integrity of delicate electrode structures throughout the HSR™ process.

## Methods

### Electrode fabrication via femtosecond laser hierarchical surface restructuring (HSR™)

The electrode arrays used in this study are fully-assembled paddle-style arrays designed for spinal cord stimulation applications. Each array consists of 16 platinum–iridium (Pt-10Ir) electrode contacts, with an individual contact surface area of approximately 12.4 mm². All surface modification via HSR™ was performed directly on these pre-assembled electrode arrays, without any alteration to the underlying manufacturing process or device architecture.

HSR™ laser processing was performed using Tescan FemtoChisel (Storrs, CT, USA). The laser source used in these experiments was a Monaco 1035 (Coherent, Santa Clara, CA, USA), that generates 257 fs pulses with a central wavelength of 1035 nm and an initial beam diameter of 2.7 mm. A telecentric, f-theta lens with a focal length of 70 mm focuses the beam at the focal point. The beam is deflected and targeted via galvo scan head. The experiments were performed in an ambient air environment. Targeting and alignment of the pattern to the electrodes was done via a digital microscope Arrays were mounted on a porous, ceramic vacuum chuck with three-dimensional stage controls and were leveled with the focal plane of the scan-head using a Confocal Displacement Sensor.

Because of the curved profile and asymmetrical thickness of the paddle arrays, the device could not form an airtight seal with the flat surface of the vacuum chuck and therefore could not be securely mounted using standard fixturing methods. To address this, a custom adapter was designed to conform to the back side of the paddle array, ensuring mechanical stability, proper vacuum sealing, and level electrode mounting during laser processing. This adapter allowed consistent and reproducible mounting of the array on the laser stage while preserving the intended orientation and curvature of the electrode contacts.

Preliminary testing revealed that the soft polymer insulation of the paddle arrays deformed under the force of the CO_2_ snow stream, resulting in slight shifts during processing and loss of alignment precision. To address this issue, the custom adapter was designed to also accommodate a clamping mechanism. This clamp securely holds the electrode array in place during laser restructuring and CO_2_ snow processing, ensuring positional stability, consistent laser focus, and precise surface modification across all electrode contacts.

To account for the variable topology of each electrode contact, the confocal sensor was used to generate a coarse height map of the surface. The area designated for restructuring was divided into 15 equally sized subdivisions (arranged in a 5 × 3 grid), and the z-height was measured at the center of each subdivision. Rather than processing the entire contact in a single lasering pass, the restructuring process was carried out one subdivision at a time. For each tile, the stage height was dynamically adjusted to bring the local surface into optimal focus under the laser, ensuring consistent energy delivery and restructuring fidelity across the full electrode contact.

This method ensured the laser remained in focus across the entire surface of the electrode contact. Although generating the height map adds approximately 25 seconds to the overall laser processing time, the resolution and duration can be substantially reduced by decreasing the number of subdivisions. In this study, 15 subdivisions were used to provide a margin of safety, particularly because some of the electrode surfaces exhibited minor warping or inconsistencies, likely due to more frequent and intensive handling. For electrodes with more level surfaces, fewer subdivisions would be sufficient.

Although electrode performance is influenced by the complex interplay of multiple laser parameters, this study—along with prior investigations—primarily focuses on laser fluence. Fluence serves as a unifying parameter because it encapsulates the effects of other variables such as laser pulse energy, spot size, and repetition rate, making it a widely applicable metric across various laser systems. Fluence is defined as the laser energy delivered per unit area, as shown in Eq. (1). Fluence provides a consistent basis for comparing results and optimizing processing conditions.


$$\begin{array}{c} Fluence\;\left(\frac{J}{{cm}^{\text{2}}}\right)=\frac{Energy\;per\;Pulse\;(J)}{Laser\;Spot\;Size\;\left({cm}^{\text{2}}\right)} \end{array}$$ (1)

Because the electrodes are embedded within soft polymer insulation, it is critical to maximize the usable electrode surface area while minimizing the risk of damaging surrounding insulation or adjacent components. To mitigate unintended thermal or mechanical effects caused by potential misalignments during laser targeting, the restructured area is intentionally recessed inward from the edge of the electrode. This built-in safety margin helps ensure that restructuring remains confined to the intended electrode area without compromising insulation integrity.

Using this approach, approximately 86% of the electrode contact surface was targeted. A narrow 0.1 mm border of unaltered material was intentionally left around the periphery to act as the protective buffer, minimizing the risk of damaging the adjacent polymer insulation. In addition to spatial control, laser fluence must also be carefully optimized to avoid unintended thermal effects, such as localized melting or deformation of the insulation, as previously discussed. To this end, three different fluence levels were tested: 3.86 J/cm², 5.96 J/cm², and 8.63 J/cm², to determine the upper limit of laser fluence. For each fluence, four channels of the electrode array were restructured, while four of the 16 total channels were left unprocessed as controls. Scanning electron microscopy (SEM) micrographs were taken before and after processing to evaluate surface modification and to ensure that none of the fluence levels caused unintended damage to either the electrode surface or the surrounding insulation.

### Surface and microstructural characterization

The morphology and microstructures present on the electrode surfaces were qualitatively examined with a scanning electron microscope (SEM) (ZEISS, Oberkochen, Germany), which provided high-resolution micrographs of nanoscale surface features.

### Electrochemical characterization

The electrochemical performance of the electrodes was evaluated using cyclic voltammetry (CV) and electrochemical impedance spectroscopy (EIS) to determine total charge storage capacity (CSC_t_), impedance, and specific capacitance (SC) [13,29,31]. CSC_t_ was calculated and normalized using the formula in Eq. (2), while SC was derived by fitting the EIS data to a Randles circuit model and normalizing for the electrode area. All CSC_t_, specific capacitance, and impedance values are reported as an average of the four corresponding electrode contacts (n = 4), with error bars representing their standard deviation. All additional CV and EIS experimental procedures and details are consistent with those described in previous publications [29,31].


$$\begin{array}{c} {CSC}_{t}=\frac{\;\text{1}}{GSA}\int Idt \end{array}$$ (2)

## Results and discussion

### Morphology and microstructure

**Fig 2:**
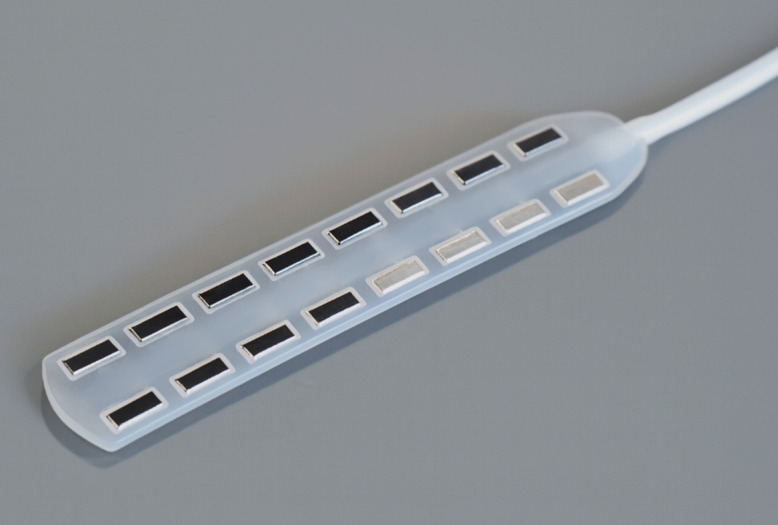
Photograph of a restructured paddle-type electrode array used for spinal cord stimulation (SCS), with three sets of four channels restructured at different laser fluences. From left to right, top to bottom: 4x 8.63 J/cm², 4x 5.96 J/cm², 4x 3.86 J/cm², and 4x unrestructured. Image used with permission and courtesy of ONWARD Medical. The central region of the electrode array, showing the connecting wires to the backside of the electrode contacts, has been intentionally blurred at the request of ONWARD Medical for proprietary reasons. This modification does not affect the objectives of this study, the interpretation of the data, or the conclusions presented in this work.

The electrode contacts on the paddle electrode arrays were successfully restructured, producing a periodic, hierarchical surface across approximately 86% of the total electrode area. This restructuring coverage was achieved while maintaining an unmodified peripheral boundary to protect adjacent insulation from thermal or mechanical damage. A representative photograph of one of the paddle arrays restructured at three laser fluence levels—3.86 J/cm², 5.96 J/cm², and 8.63 J/cm²—is shown in Fig 2. In the same array, four electrode contacts were intentionally left unrestructured to serve as controls. The image clearly demonstrates the transformation induced by the laser restructuring process and highlights the pronounced contrast between the hierarchically restructured electrode contacts (darker contacts) and the unrestructured control electrodes (lighter contacts).

**Fig 3:**
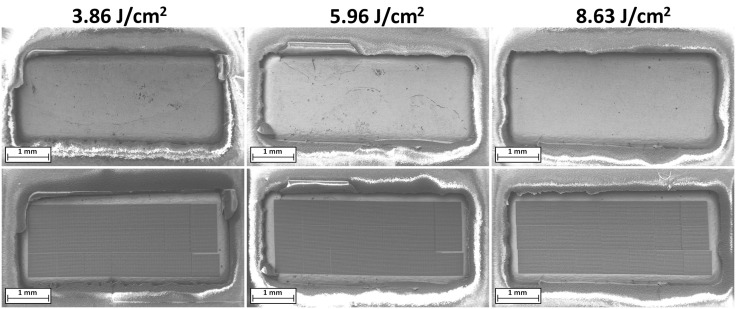
SEM micrographs of paddle electrode contacts, before (top) and after (bottom) HSR™. Comparison of the edges of the insulation surrounding electrode contact shows no significant changes or damage as a result of laser restructuring.

SEM micrographs taken before and after restructuring at each of the three fluence levels (Fig 3) confirmed that the laser restructuring process did not result in any physical damage to the electrode contacts’ surfaces or the adjacent polymer insulation. Across all tested fluences, the restructuring consistently produced hierarchical surface features without signs of melting, delamination, or collateral damage. The polymer insulation remained intact and unaffected, indicating that the applied laser parameters and the recessed design of the restructured area effectively mitigated risks associated with stray laser energy or thermal conduction. The lack of any damage at the fluences tested also indicates that higher fluences may be safely utilized, providing even greater improvements to electrochemical performance without collateral damage [29,30].

The restructuring process without CO_2_-snow-assisted processing required approximately 14 seconds per mm² of treated surface area, including the time for height mapping. This translated to a total processing time of 130 seconds per electrode channel. When tandem CO_2_-snow-assisted processing was applied, the time increased slightly to 16 seconds per mm², resulting in 150 seconds per electrode channel. But as noted earlier, the overall processing time can be reduced by decreasing the resolution of the height map. The number of subdivisions used in the mapping process can be optimized based on the uniformity of the electrode surface topology, allowing for faster processing without compromising targeting accuracy on smoother electrodes.

Preliminary experiments demonstrated that reducing the resolution of the height map to a 2 × 3 subdivision grid lowered laser processing time to ~ 11 seconds per mm², or approximately 100 seconds per electrode. This improvement is primarily due to the reduced time required to generate the height map and the fewer number of stage movements needed during the HSR™ process. By minimizing mechanical motions and computational load, lower-resolution mapping offers a practical means of accelerating throughput while still maintaining sufficient targeting accuracy for consistent electrode restructuring.

As shown in Fig 3, a narrow bar of unrestructured area appears along the bottom-right edge of some restructured electrodes, representing a slight break in the HSR™ pattern. This recurring gap is attributed to a software error caused by the computational intensity of rendering high-resolution hierarchical surface patterns. The affected area is minimal relative to the total electrode surface area and does not materially impact the electrochemical performance of the array. Ongoing improvements to the control software aim to eliminate this anomaly in future iterations.

**Fig 4:**
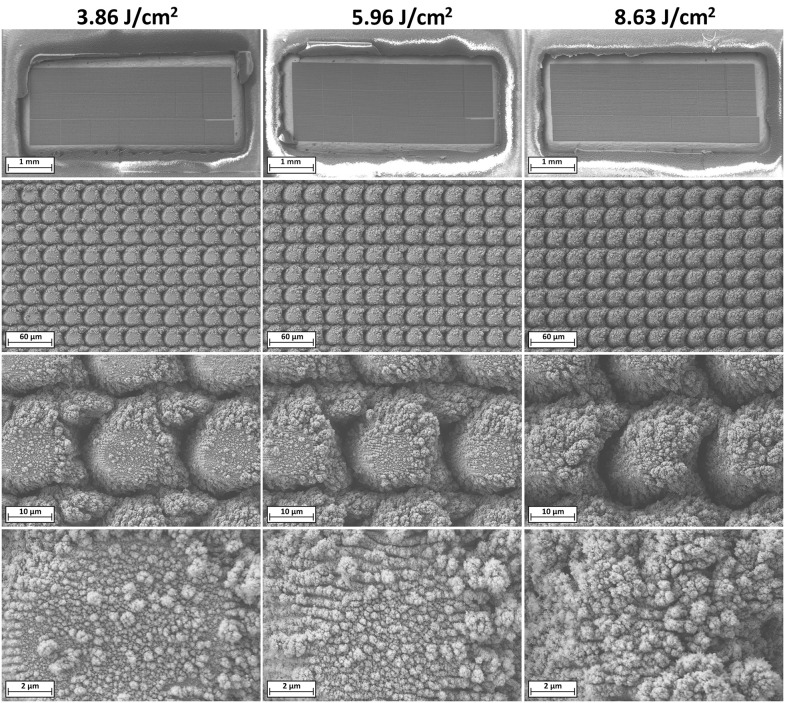
SEM micrographs of HSR™ electrodes fabricated without CO_2_-snow-assisted processing at laser fluences of (left to right) 3.86, 5.96, and 8.63 J/cm^2^ and magnifications of (top to bottom) 70x, 1kx, 5kx, 20kx to capture macro, micro, and nano-scale morphological features.

Fig 4 provides a magnified view of the electrode surface morphology achieved through the HSR™ process at each tested fluence level, excluding CO_2_-snow-assisted processing. At the coarse scale, the hierarchical surface structures exhibit densely packed, circular, pillar-like features, typically ranging from 20 to 30 µm in diameter, depending on the fluence. These microstructures contribute significantly to the increased surface roughness and ESA of the treated electrodes. The centers of these pillar-like structures appear to be more lightly restructured compared to their perimeters and the surrounding valleys. This is primarily due to their exposure to the lower-intensity edges of the laser beam profile. These beam tails lack sufficient energy density to induce complete restructuring, resulting in less pronounced surface modification and restructuring. As the laser fluence and the energy of the beam tail increase, the lightly restructured central regions of the pillars begin to exhibit progressively more pronounced restructuring, becoming increasingly uniform with the surrounding perimeter features. This is attributed to the higher energy density in the beam tails, which gradually reaches the threshold necessary for effective surface restructuring. Concurrently, the valleys between adjacent pillars deepen and widen with increasing fluence, as the higher-energy laser pulses ablate more material. Valley depths range from approximately 4–10 µm, correlating with the increasing intensity of material removal at elevated fluences. At the nanoscale, the surface of each pillar is densely covered with high surface area nanostructures, which are most clearly resolved in the higher magnification SEM micrographs. These nanostructures contribute significantly to the overall surface roughness and electrochemically active surface area (ESA). As the laser fluence increases, both the frequency and spatial density of these nanostructures increase, progressively covering a larger fraction of the electrode surface. This enhanced nanoscale texturing adds substantial surface area and complexity to the hierarchical architecture, further augmenting the electrode’s electrochemical performance.

**Fig 5:**
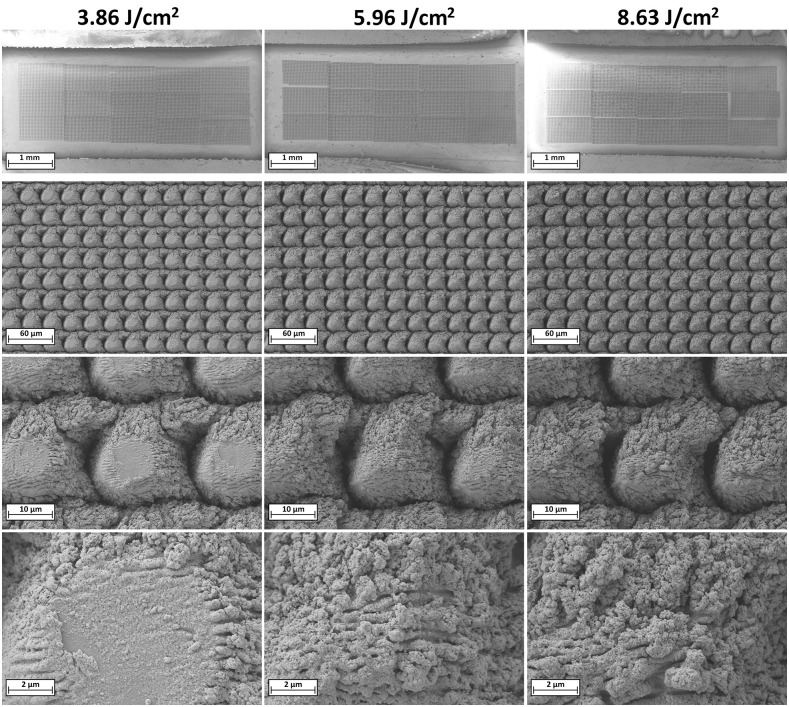
SEM micrographs of HSR™ electrodes fabricated with tandem CO_2_-snow-assisted processing at laser fluences of (left to right) 3.86, 5.96, and 8.63 J/cm^2^ and magnifications of (top to bottom) 70x, 1kx, 5kx, 20kx to capture macro, micro, and nano-scale morphological features.

Fig 5 presents electrode contacts restructured at the same set of fluences but with the addition of tandem CO_2_-snow-assisted processing. While the overall macrostructure and underlying hierarchical features remain consistent with those seen in standard HSR™ processing, the introduction of the high-velocity CO_2_ jet leads to several notable differences in morphology. At higher magnifications (5,000× and 20,000×), the electrode surfaces appear smoother and more uniform, suggesting that the CO_2_ stream influences the surface while it is in a thermally excited or transiently softened state during laser interaction.

This effect facilitates the removal of loosely bound particulates, promoting a more refined micro- and nanoscale texture while preserving the underlying electrode macrostructure. The minimally abrasive nature of the process arises from a thin film of liquid CO_2_ that forms on the surface, effectively cushioning the impact energy of the dry ice particles during impingement on the electrode surface [33]. The force generated by the high-velocity CO_2_ jet stream may contribute to a smoothing of the surface texture, particularly by suppressing the formation of finer nanostructures during laser exposure. The result is a hierarchical structure with reduced surface roughness and potentially enhanced stability, without compromising the overall increase in ESA. This smoothing effect is especially pronounced on the south-facing surfaces of the pillar structures, which are oriented directly toward the CO_2_ nozzle. These regions appear to experience greater aerodynamic drag during processing, resulting in both a visibly smoother texture and a notable flattening of the south face. Consequently, the morphology of the pillars shifts from a more circular footprint to an asymmetrical or slightly oblong shape.

A noticeable reduction in surface nanostructures is observed across the restructured electrodes processed with tandem CO_2_-snow-assisted processing. These nanostructures are primarily the result of redeposited and loosely bound debris that accumulate during laser processing. The drag force exerted by the high-velocity CO_2_ stream effectively inhibits their redeposition and removes weakly adhered surface particulates. As a result, the electrode surface appears cleaner and more uniform, with significantly fewer impermanent nano-scale features. While these impermanent nanostructures exhibit high surface area and initially contribute to enhanced electrochemical performance, they are mechanically unstable and prone to detachment during electrochemical cycling. This detachment results in performance degradation over time, as the loosely bound nanoparticles are gradually dislodged during device operation. In a previous study, it was shown that tandem CO_2_-snow-assisted processing preemptively removes these unstable nanostructures, thereby enhancing morphological integrity and promoting long-term electrochemical stability.

### Electrochemical performance

**Fig 6:**
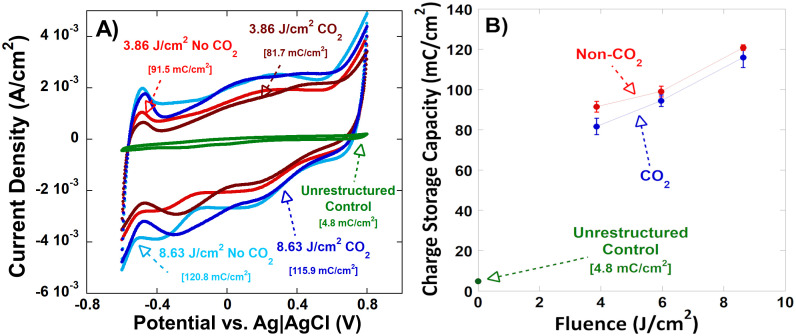
A) Cyclic voltammograms of electrodes restructured at 3.86 J/cm^2^ and 8.63 J/cm^2^, with and without tandem CO_2_-snow-assisted processing, compared to an unrestructured control electrode. Voltammograms are taken from −0.6V to 0.8V, against an Ag/AgCl electrode. Labels indicate corresponding total charge storage capacity (CSCt) for each voltammogram. **B)** Total charge storage capacity (CSCt) for electrodes restructured at 3.86 J/cm2, 5.96 J/cm2, and 8.63 J/cm2, with and without CO_2_-snow-assisted processing, and an unrestructured control electrode.

To evaluate the electrochemical performance of HSR™-treated electrodes in the arrays, cyclic voltammetry (CV) was performed across all laser fluence levels and the results were compared against performance of baseline (unrestructured) control electrodes, as shown in Fig 6a. The area enclosed by each CV curve corresponds to the total charge storage capacity (CSC_t_), with larger enclosed areas indicating enhanced electrochemical performance. The cyclic voltammograms of restructured electrodes display a substantial increase in area compared to the unrestructured controls, indicating enhanced electrochemical performance. This increase in area scales with laser fluence, reflecting the greater ESA generated through surface restructuring. This improvement in performance was quantified by integrating the current over time to calculate CSC_t_ for each tested electrode contact, with and without tandem CO_2_-snow-assisted processing. The resulting CSC_t_ values versus laser fluence are plotted in Fig 6b. CSC_t_ increases positively and approximately linearly with laser fluence within the tested range. However, prior studies have shown that across a broader range of fluences, this relationship tends to follow a more logarithmic trend, due to diminishing returns in surface area and roughness [29,30]. The fluences tested in this study are relatively low enough, that this plateau in surface area and subsequently, electrochemical performance, does manifest, making the relationship between performance and fluence appear linear.

Without tandem CO_2_-snow-assisted processing, the tested fluences resulted in CSC_t_ values ranging from approximately 90–120 mC/cm², representing an over 2400% increase in electrochemical performance relative to the unrestructured baseline electrodes (4.8 mC/cm²). When tandem CO_2_-snow-assisted processing was applied, the same trend in CSC_t_ with respect to fluence was observed, but overall values were slightly lower. With CO_2_-snow-assisted processing, CSC_t_ ranged from approximately 80–116 mC/cm², equating to an over 2300% improvement. This modest reduction in performance is attributed to the smoother surface texture induced by the CO_2_ jet stream, which reduces the presence of high-surface-area nanostructures that contribute to CSC_t_ but are prone to detachment during use.

**Fig 7:**
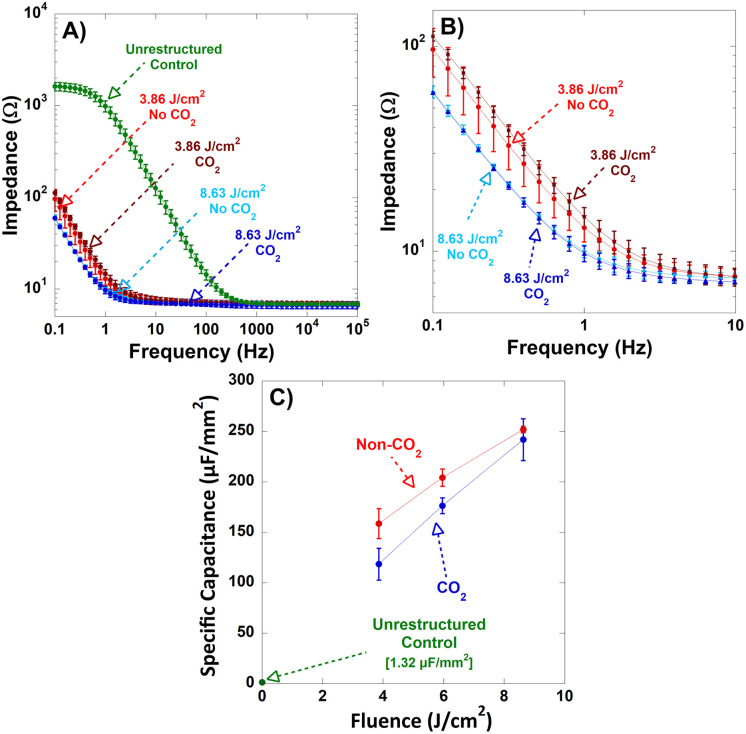
A) Bode plots of impedance magnitude as a function of frequency (plotted in the 0.1-10^4^ Hz frequency range) for unrestructured electrodes, and electrodes restructured at 3.86 J/cm^2^ and 8.63 J/cm^2^, with and without tandem CO_2_-snow-assisted processing, and an unrestructured control electrode. **B)** Bode plots of impedance magnitude as a function of frequency with a magnified scale, for improved legibility of the impedances of restructured electrodes. **C)** Specific capacitance for electrodes restructured at 3.86 J/cm^2^, 5.96 J/cm^2^, and 8.63 J/cm^2^, with and without CO_2_-snow-assisted processing, and an unrestructured control electrode.

Electrochemical impedance spectroscopy (EIS) was used to characterize the impedance behavior of the electrodes across a frequency range of 0.1 Hz to 10⁴ Hz, with the results displayed as Bode plots in Fig 7a, and magnified in Fig 7b. Across this entire range, the restructured electrodes exhibited a marked reduction in impedance relative to the unrestructured controls. Notably, at 1 kHz, which is a standard benchmark frequency for neural stimulation applications, all restructured electrodes converged to exhibit comparable impedance values. This indicates that laser restructuring yields consistently low-impedance surfaces suitable for neural stimulation but enables a wider range of low-impedance operating conditions.

To further quantify the electrochemical behavior of the electrodes, EIS data were fitted using a Randles equivalent circuit model to extract specific capacitance values, as shown in Fig 7b. The trend in specific capacitance mirrors that of CSCₜ, increasing approximately linearly with laser fluence across the tested range. Without CO_2_-snow-assisted processing, specific capacitance values ranged from approximately 155–255 µF/mm², representing an extraordinary enhancement of up to 19,000% compared to 1.32 µF/mm² in unrestructured control electrodes. With CO_2_-snow-assisted processing, the overall trend remained consistent, but the capacitance decreased by an average of approximately 12%, yielding a range of approximately 118–241 µF/mm². This reduction corresponds to the smoother surface morphology resulting from CO_2_-snow-assisted processing and removal of unstable nanostructures. As observed in earlier studies, the relationship between fluence and capacitance follows a more logarithmic trajectory when extended to a broader range of fluences [29,30].

## Concluding remarks

Femtosecond laser Hierarchical Surface Restructuring (HSR™) has previously been shown to be a promising surface modification technology for advancing the development of next-generation neural interfacing electrodes. In this work, we developed a workflow for the first time to adapt the HSR™ technology for restructuring and surface modification of fully assembled Pt-10Ir paddle arrays used for spinal cord stimulation, without any collateral damage to other array components, caused by stray laser energy or thermal conduction. The tuneability of the technique was demonstrated by utilizing a range of laser fluences that enable substantial improvements in electrodes’ total charge storage capacity and specific capacitance, compared to unaltered control electrodes. Because no collateral damage was observed at the highest fluence tested in this study, this indicates higher fluences may be safely utilized, corresponding to even greater benefits to electrochemical performance. In-operando CO_2_-snow-assisted restructuring was also demonstrated to be compatible with prefabricated assembled electrode arrays, yielding more stable electrode surfaces, at the cost of a slight decrease in electrochemical performance. These findings indicate that the advantages offered by HSR™ technology with CO_2_-snow-assisted processing to electrode performance and morphology, such as improved signal-to-noise-ratio, energy efficiency, impedance, and surface stability, are compatible with existing neural interfacing devices. With some adaptations for a device’s geometric and material constraints, the HSR™ technology has the potential to be seamlessly integrated into existing assembly lines as a post-process, without any changes to the upstream manufacturing processes. This versatility represents a significant advancement towards the widespread adoption of HSR™ technology and makes the technique of interest to long-term implantable device manufacturers looking to enhance the performance of electrodes, and miniaturize neural interfacing devices in a cost-effective manner. This work lowers the barrier for industrial implementation of HSR™ technology by simplifying its integration into existing manufacturing workflows as a post-fabrication surface modification step, eliminating the need for costly process changes or specialized equipment.

Long-term in-vitro stimulation studies are currently underway to evaluate the electrochemical stability and durability of HSR™ electrode surfaces under prolonged current stimulation, with direct comparison to commercially established high-performance electrode surface technologies such as titanium nitride (TiN) and iridium oxide (IrO_2_) coatings. These studies are intended to provide a rigorous and necessary validation of HSR™ performance relative to state-of-the-art electrode materials under controlled, application-relevant electrochemical conditions. While active neural recording and stimulation in biological environments remain an important translational objective, the present and ongoing efforts prioritize establishing the fundamental electrochemical robustness, manufacturability, and long-term stability of the HSR™ platform as a prerequisite for subsequent in-vivo neurostimulation and neural recording investigations.
